# Structural and dynamic vascular factors are not related to apathy over time: time to rethink the vascular apathy hypothesis?

**DOI:** 10.1192/j.eurpsy.2025.530

**Published:** 2025-08-26

**Authors:** A. S. Bertens, K. Moret, J. Van der Grond, R. Poortvliet, N. Rius Ottenheim

**Affiliations:** 1 Psychiatry, Leiden University Medical Center; 2 Old Age Psychiatry, Rivierduinen GGZ Mental Health Services; 3 Faculty of Social Sciences, Leiden University; 4 Radiology; 5 Public Health and primary care, Leiden University Medical Center, Leiden, Netherlands

## Abstract

**Introduction:**

Apathy frequently occurs in older persons but its aetiology is not understood as of yet. One hypothesis, the vascular hypothesis of apathy, suggests a link between apathy and vascular factors such as cerebral small vessel disease (CSVD), that may cause lesions in the reward network of the brain. In previous studies, a cross sectional association was found between lower blood pressure (BP) and symptoms of apathy in older persons with more CSVD, potentially through reduced cerebral blood flow (CBF). However, longitudinal studies on associations between vascular factors and apathy are scarce.

**Objectives:**

We investigated whether in older persons, structural and dynamic vascular factors are associated with apathy symptoms over time. We hypothesized that a higher burden of CSVD, a lower BP and lower CBF, would be related to an increase in symptoms of apathy.

**Methods:**

This study is a longitudinal cohort study involving community-dwelling older participants of the Discontinuation of Antihypertensive Treatment in the Elderly (DANTE) Study, all using antihypertensive treatment and with mild cognitive deficits. At baseline BP was measured and apathy was assessed with the Apathy Scale (AS, range 0-42) at baseline and after four years of follow-up (n=178). Additionally, a baseline MRI sub study (n=109) was conducted to measure CSVD and CBF. Univariate and multivariate linear regression analyses were performed using CSVD, BP and CBF as determinants and the change in Apathy Scale score over time as an outcome.

**Results:**

Mean age of the population was 80 years (SD 4) and 63% was female. In the MRI sub study, no significant association was found between the summary CSVD scores (β (95% CI)=0.018(-1.089-1.125), *p*=.975) or it’s separate features; WMH (β(95% CI)=0.012(-0.011-0.035), *p*=.318), CMB (β (95% CI)=-0.017(-0.605-0.572), *p*=.956), lacunar infarctions (β(95% CI)=-0.413(-1.266-0.440), *p*=.339), and a change in Apathy Scale score. Additionally, no significant association between the dynamic vascular factors; CBF (β(95% CI)=-0.029(-0.152-0.094), *p*=.640), systolic BP (β (95% CI)=-0.019 (-0.056-0.018), *p=.*310) and diastolic BP (β(95% CI)=-0.029(-0.099-0.042), *p*=.425), and change in Apathy Scale score was found. The multivariate linear regression model, which incorporated all the structural and dynamic vascular parameters, age, and gender, was not significant.

**Conclusions:**

In older persons with mild cognitive deficits, structural and dynamic vascular factors were not associated with symptoms of apathy after four years of follow-up. Our findings thus do not support a ‘vascular apathy hypothesis’. Potentially, other factors such as life style factors confound the cross sectional association between vascular factors and apathy, or different apathy syndromes may have different aetiologies. Larger studies with less baseline vascular burden, are needed to confirm our results.

**Disclosure of Interest:**

None Declared

